# Mitochondrial DNA Sequence Variation and Haplogroup Distribution in Chinese Patients with LHON and m.14484T>C

**DOI:** 10.1371/journal.pone.0013426

**Published:** 2010-10-18

**Authors:** Dandan Yu, Xiaoyun Jia, A-Mei Zhang, Shiqiang Li, Yang Zou, Qingjiong Zhang, Yong-Gang Yao

**Affiliations:** 1 Key Laboratory of Animal Models and Human Disease Mechanisms, Kunming Institute of Zoology, Chinese Academy of Sciences, Kunming, China; 2 State Key Laboratory of Ophthalmology, Zhongshan Ophthalmic Center, Sun Yat-sen University, Guangzhou, China; 3 Graduate School of the Chinese Academy of Sciences, Beijing, China; University of Florida, United States of America

## Abstract

**Background:**

Leber hereditary optic neuropathy (LHON, MIM 535000) is one of the most common mitochondrial genetic disorders caused by three primary mtDNA mutations (m.3460G>A, m.11778G>A and m. 14484T>C). The clinical expression of LHON is affected by many additional factors, e.g. mtDNA background, nuclear genes, and environmental factors. Hitherto, there is no comprehensive study of Chinese LHON patients with m.14484T>C.

**Methodology/Principal Findings:**

In this study, we analyzed the mtDNA sequence variations and haplogroup distribution pattern of the largest number of Chinese LHON patients with m.14484T>C to date. We first determined the complete mtDNA sequences in eleven LHON probands with m.14484T>C, to discern the potentially pathogenic mutations that co-segregate with m.14484T>C. We then dissected the matrilineal structure of 52 patients with m.14484T>C (including 14 from unrelated families and 38 sporadic cases) and compared it with the reported Han Chinese from general populations. Complete mtDNA sequencing showed that the eleven matrilines belonged to nine haplogroups including Y2, C4a, M8a, M10a1a, G1a1, G2a1, G2b2, D5a2a1, and D5c. We did not identify putatively pathogenic mutation that was co-segregated with m.14484T>C in these lineages based on the evolutionary analysis. Compared with the reported Han Chinese from general populations, the LHON patients with m.14484T>C had significantly higher frequency of haplogroups C, G, M10, and Y, but a lower frequency of haplogroup F. Intriguingly, we also observed a lower prevalence of F lineages in LHON subjects with m.11778G>A in our previous study, suggesting that this haplogroup may enact similar role during the onset of LHON in the presence of m.14484T>C or m.11778G>A.

**Conclusions/Significance:**

Our current study provided a comprehensive profile regarding the mtDNA variation and background of Chinese patients with LHON and m.14484T>C. Matrilineal background might affect the expression of LHON in Chinese patients with m.14484T>C.

## Introduction

Leber hereditary optic neuropathy (LHON, MIM 535000) is one of the most common mitochondrial genetic disorders caused by mtDNA point mutation [1] and causes visual loss in young adults. The clinical diagnosis of LHON is mainly based on ophthalmologic features and molecular genetic testing for three primary mutations (m.3460G>A in the *MT-ND1* gene, m.11778G>A in the *MT-ND4* gene, and m. 14484T>C in the *MT-ND6* gene) [1]–[6]. Over 95% of LHON patients carries one of the three primary mutations [2], but not all individuals will develop typical symptom of LHON (viz. incomplete penetrance), which suggests that some additional factors, e.g. other mtDNA mutations, mtDNA background, nuclear genes, and environmental factors, may be involved in the phenotypic expression, severity, and recovery of this disorder [1], [2], [7]–[10]. Indeed, the three primary mutations are prerequisites for LHON, and many other rare LHON mutations are discovered continuously (c.f. http://www.mitomap.org/MITOMAP/MutationsLHON). For instance, mutation m.3635G>A was reported in several LHON families without the three primary mutations and appeared to be one of LHON primary mutations in Chinese families [11]–[13]. Mutations m.3736G>A (p.V144I) and m.10680G>A (p.A71T) were also among the list of pathogenic mutations for Chinese patients with LHON or suspected LHON [14].

Among these factors that affect the clinical expression of LHON, mtDNA background/haplogroup, which was formed during the migration of the ancestor of modern human across world, would have an influence on the expression of LHON [9], [15]. Those early reports for secondary mutations for LHON in patients from Europe or of European origin, e.g. m.4216T>C, m.13708G>A, and m.15257G>A [16]–[18] were actually haplogroup-specific variants for haplogroup J2. Because of the continent-specific distribution of mtDNA haplogroups, there is a remarkable difference of the haplogroups that had an influence on the expression of LHON between the Western European patients and East Asian patients. Haplogroups J1, J2, and K increased the risk of blindness in LHON patients with m.14484T>C, m.11778G>A, and m.3640G>A, respectively, whereas haplogroup H had a protective role in LHON patients with m.11778G>A in European LHON families [9]. By analyzing the LHON penetrance pattern in Chinese families with m.11778G>A, we recently found that haplogroup M7b1′2 significantly increased the risk of visual failure and M8a had a protective effect [15]. Experimental data on LHON hybrid cell models harboring the three primary LHON mutations showed that specific mtDNA haplogroup modulated OXPHOS assembly kinetics [19] or increased the sensitivity to environmental toxicity [20]. These results further supported the notion that mtDNA haplogroups played an important role in the onset of LHON.

We identified 537 Chinese LHON patients with one of the three primary mutations (m.3460G>A, m.11778G>A, or m. 14484T>C) during our recent campaign for comprehensive mutation screening for this disease in a large cohort of patients (N = 1,626) with clinical feature of LHON or suspected with LHON [15], [21]. Among these LHON patients confirmed by genetic diagnosis, we identified 52 families/singleton cases harbored mutation m.14484T>C. In this study, we presented our analyses for mtDNA sequence variation and haplogroup classification of these mtDNAs with m.14484T>C. We first selected eleven LHON probands and determined the complete mtDNA sequences, to discern potential secondary mtDNA mutation(s) or co-segregation of pathogenic mutation(s) in these cases. We then dissected the matrilineal genetic structure of all LHON patients with m.14484T>C and compared to that of the general Han Chinese. The direct comparison of each haplogroup in LHON patients and general Han Chinese showed that there was a strikingly lower prevalence of haplogroup F in the case group.

## Materials and Methods

### Ethics statement

Written informed consents conforming to the tenets of the Declaration of Helsinki were obtained from each participant prior to the study. The institutional review boards of the Zhongshan Ophthalmic Center and the Kunming Institute of Zoology approved this study.

### Patients

Fifty-two patients with LHON and m.14484T>C were collected from different provinces in China, which were identified from 537 patients who were genetically diagnosed as LHON according to the presence of one of the three LHON primary mutations. The presence of mutation m.14484T>C in these patients was confirmed at the Kunming Institute of Zoology and at the Zhongshan Opthalmic Center, independently, by using the allele-specific PCR, SSCP (single strand conformation polymorphism), and/or sequencing [22], [23]. Among them, 14 probands were from pedigrees with a family history of LHON and 38 cases were sporadic. Note that we defined the proband from a family with only one patient (according to the accessible pedigree information) or with unclear family history as sporadic. This definition for sporadic case also includes the family with several asymptomatic carriers of m.14484T>C but with only one affected member (viz. the proband).

### mtDNA sequencing

Genomic DNA was extracted from peripheral blood by the standard phenol-chloroform method. The noncoding control-region sequences were analyzed in 52 patients as described in our previous study [15], [24]. Coding-region mutation motifs (e.g. m.5178C>A [MT-ND2: p.L237M; MIM 516001] for haplogroup D, recognized by -5176 *Alu*I) were screened to further solidify the haplogroup status for some mtDNAs. The entire mtDNA sequences of eleven probands were amplified by four overlapping primers according to our previous study [25]. PCR products were purified on spin columns (Tiangen Biotech Co., Beijing, China) and directly sequenced by using the reported inner primers [25], [26] and BigDye Terminator v3.1 Cycle Sequencing Kit (Applied Biosystems, CA, USA) on a 3730 DNA sequencer (Applied Biosystems).

### Data analysis

We evaluated the penetrance rate of LHON in the pedigrees based on our previous criterion [14]. Namely, we excluded the following family members from the analysis: (a) the first generation, (b) spouses of the matrilineal members, and (c) children of the male member in each family. Sequence variations in each proband mtDNA sequence were scored relative to the revised Cambridge Reference Sequence (rCRS) [27]. We followed the recently updated version of East Asian mtDNA tree [28] and the PhyloTree.org database (http://www.phylotree.org) [mtDNA tree Build 10; 10 Aug 2009] to classify each sample [29]. Potential sequence abnormalities that were identified by using the phylogenetic approach [30], [31], as performed manually and/or automatically by the MitoTool (http://www.mitotool.org; this software was designed by ourselves), were rechecked for the original sequencing electropherograms or resequencing. We defined the uniqueness of mtDNA variant(s) in certain matriline by an exhaustive database search following the available guidelines [32]. A schematic tree was reconstructed to show the relationship among these mtDNAs. Evolutionary conservation analysis for certain mtDNA variant was performed using the same approach as described in our previous study [15], [25]. Statistical analysis was performed using the SPSS statistical package (version 11.5), and statistical significance was established at *P*<0.05.

## Results

### Prevalence and heteroplasmy of m.14484T>C in Chinese patients with LHON

Among 537 Chinese patients with LHON and one of the three primary LHON mutations, 52 subjects were confirmed to harbor m.14484T>C. The prevalence of m.14484T>C in all 537 LHON patients (9.7%, 52/537) was much lower than that of European patients, in which 20.8% (33/159) of LHON patients were caused by this mutation [9]. Note that the presence of m.14484T>C in the general Han Chinese populations was very rare and we detected no individual carrying m.14484T>C in 1571 subjects (0/1571) in our recent study to screen the three LHON primary mutations in the general Chinese population [22].

**Figure 1:**
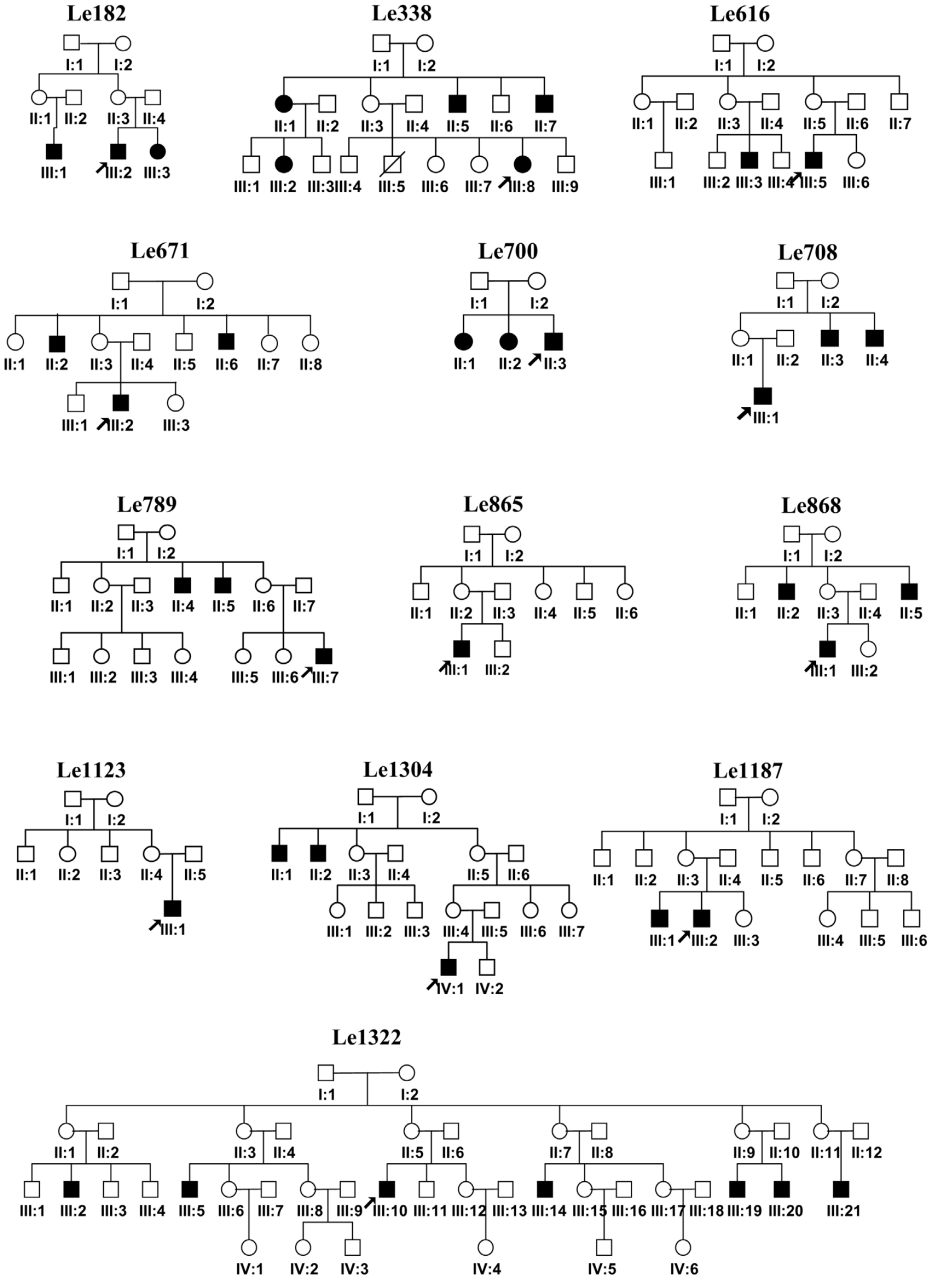
Pedigree information for Chinese LHON families with m.14484T>C. Affected individuals are marked by *filled symbols*. *Arrows* indicate the probands that were analyzed for mtDNA sequence variations in this study. Three families with a disease history but lacked of follow-up message (Le405, Le559 and Le691) were not included here for analysis. Probands Le865 and Le1123 were considered as sporadic cases according to our definition for being sporadic.

Among all Chinese LHON patients with m.14484T>C, 14 probands (14/52, 26.9%) were self-reported to have a family history of the disease and 38 cases were sporadic according to our definition for sporadic case (cf. Materials and Methods section). Unfortunately, we were unable to collect the detailed pedigree information for three families (Le405, Le559, and Le691) because of loss of follow-up message: we failed to get in touch with them according to the home address in their previous medical record. We then restricted our analysis to the remaining 11 families to estimate the affected male-to-female ratio (Fig. 1). Apparently, the affected male-to-female ratio in these families with m.14484T>C (5.2∶1, [31/6]) was substantially higher than those of families with m.11778G>A (2.38∶1, [436/183]) and m.3460G>A (4∶1, [8/2]) [15], [21]. The penetrance of LHON in these 11 families varied differently, despite a fact that some pedigrees were rather small and this would lead to a biased estimation for the penetrance rate (Fig. 1). The overall penetrance of LHON in Chinese families with m.14484T>C (31.9% [37/116]) was within the range that was observed for Chinese families with m.3460G>A (25.6% [10/39]) [21] and m.11778G>A (33.3% [619/1859]) [15]. There were only 3 cases (Le1, Le405, and Le567) with a heteroplasmic status of m.14484T>C in all 52 probands. The frequency of the heteroplasmy of m.14484T>C in Chinese LHON patients (5.8%, [3/52]) was lower than that of European patients (36.4%, [8/22]) [33]. Again, this value should be treated with caution, as we did not screen the heteroplasmy of m.14484T>C in all normal members from these families.

### Absence of co-segregation of pathogenic mutations with m.14484T>C in Chinese families with LHON

**Figure 2:**
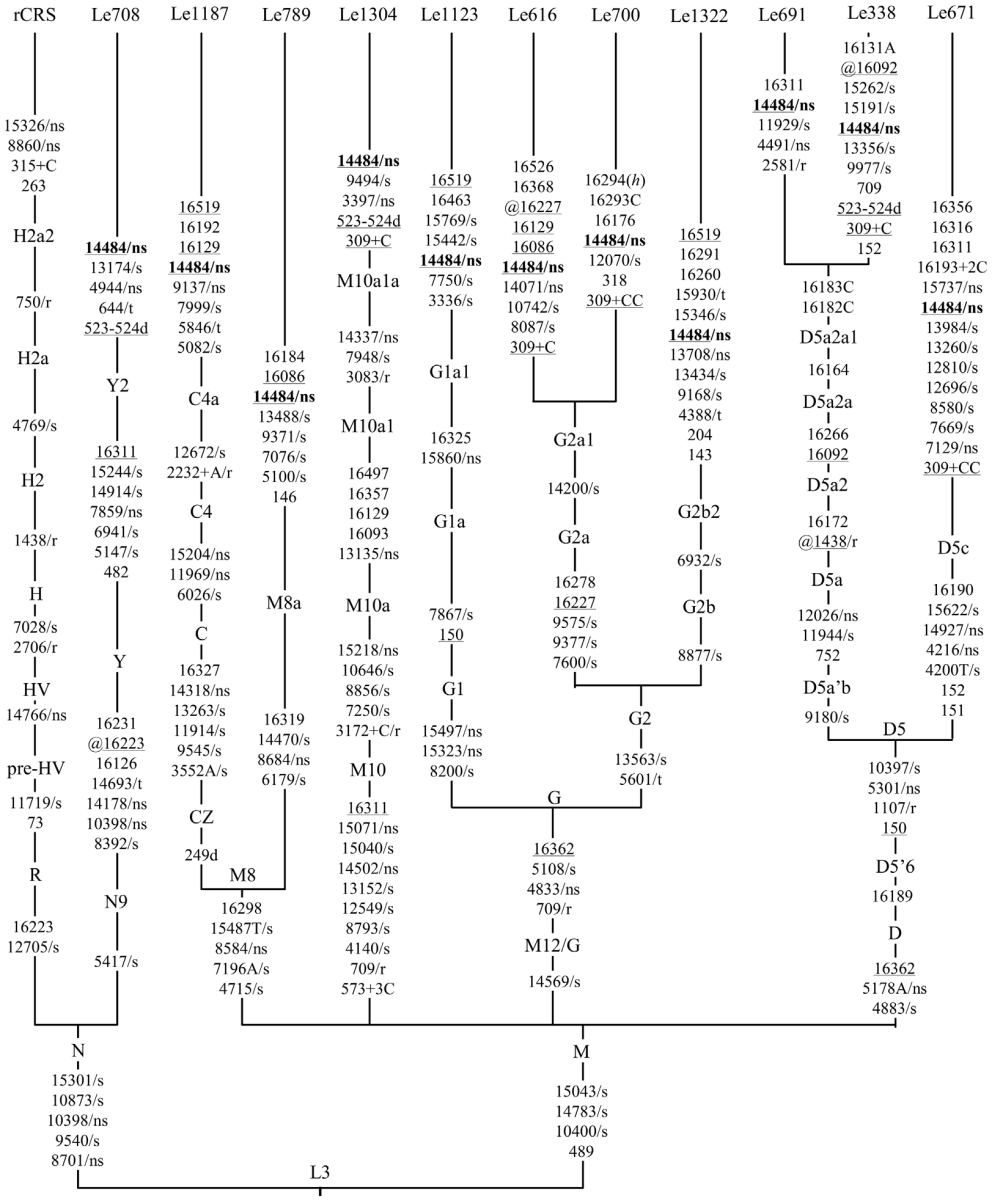
Classification tree of the entire mtDNA sequences of eleven matrilines with m.14484T>C, plus the revised Cambridge reference sequence *(rCRS)*. The order of mutations/variants on each uninterrupted branch section is arbitrary. Back variants are highlighted by the prefix “*@*”, and recurrent variants are *underlined*. Suffix ‘*+C*’ indicates an insertion of cytosine. The primary mutation m.14484T>C is in *bold*. The heteroplasmic status was marked by “(*h*)”. The synonymous and non-synonymous coding-region variants in the eleven mtDNAs are denoted by “*/s*” and “*/ns*”, respectively. Variations in the transfer RNA and the ribosomal RNA genes are denoted by “*/t*” and “*/r*”, respectively.

Previous studies have shown that there were potentially pathogenic variants co-segregated with the primary LHON mutation and enhanced the clinical expression of LHON [34], [35]. To investigate whether there are such variants in Chinese LHON families, we analyzed the complete mtDNA genomes of eleven LHON probands harboring m.14484T>C and with a family history (including one sporadic case Le1123). Probands Le182 and Le868 were not analyzed for complete mtDNA sequences, because we used up the limited genomic DNA extracted from dried blood spots on filter paper during the initial primary mutation screening and mtDNA control region sequencing. As shown in Fig. 2, these probands belonged to nine distinct mtDNA haplogroups including Y2, C4a, M8a, M10a1a, G1a1, G2a1, G2b2, D5a2a1, and D5c. There were only eight non-synonymous variants, four mt-tRNA variants, and one 16S rRNA variant in families Le708 (m.644A>G [*MT-TF*]; m.4944A>G [MT-ND2: p.I159V]), Le1187 (m.5846C>T [*MT-TY*]; m.9137T>C [MT-ATP6: p.I204T]), Le1304 (m.3397A>G [MT-ND1: p.M31V]), Le1322 (m.4388A>G [*MT-TQ*]; m.13708G>A [MT-ND5: p.A458T]; m.15930G>A [*MT-TT*]), Le671 (m.7129A>G [MT-CO1: p.Y409C]; m.15737G>A [MT-CYB: p.D331N]), Le691 (m.2581A>G [*MT-RNR2*]; m.4491G>A [MT-ND2: p.V8A]) and Le616 (m.14071A>G [MT-ND5: p.T579A]) at the twig level of the tree. These thirteen variants had been previously reported in the general populations (Table 1). Therefore, these private variants should be best categorized as polymorphisms. Family Le700 had a high penetrance of LHON (note that this pedigree was rather small and this would lead to a biased estimation for penetrance), but we failed to find any potentially pathogenic mutations that had a synergistic effect with m.14484T>C. This was quite different from the family that was described by Yang et al. [34], in which mutation m.10680G>A enhanced the expression of m.14484T>C.

**Table 1:** Private non-synonymous, mt-rRNA and mt-tRNA variants in Chinese LHON mtDNAs with m.14484T>C.

Family/subject^a^	Haplogroup	Nucleotide variant (amino acid change)	Gene	Reported (population context)^b^	Reported (disease context)^b^	Conservation c	Haplogroup-specific variant d
Le708	Y2	m.644A>G	*MT-TF*	Yes	Yes	No	Yes (M38b)
		m.4944A>G (p.I159V)	*MT-ND2*	Yes	Yes	No	No
Le1187	C4a	m.5846C>T	*MT-TY*	Yes	No	No	No
		m.9137T>C (p.I204T)	*MT-ATP6*	Yes	Yes	No	No
Le1304	M10a1a	m.3397A>G (p.M31V)	*MT-ND1*	Yes	Yes	No	Yes (C5d1a, M27c, R0a2j)
Le616	G2a1	m.14071A>G (p.T579A)	*MT-ND5*	No	Yes	No	No
Le1322	G2b2	m.4388A>G	*MT-TQ*	Yes	Yes	Yes	Yes (L3h1)
		m.13708G>A (p.A458T)	*MT-ND5*	Yes	Yes	No	Yes (M5c1, C7a1a2, M12b, etc)
		m.15930G>A	*MT-TT*	Yes	Yes	No	Yes (L0d)
Le691	D5a2	m.2581A>G	*MT-RNR2*	Yes	Yes	No	Yes (H3b)
		m.4491G>A (p.V8A)	*MT-ND2*	Yes	Yes	No	Yes (L1c1a1a2, L3c, M9)
Le671	D5c	m.7129A>G (p.Y409C)	*MT-CO1*	Yes	Yes	No	Yes (L3x2a1)
		m.15737G>A (p.D331N)	*MT-CYB*	Yes	Yes	No	No

a The complete mtDNA genomes of Le789, Le1123, Le700 and Le338 contained no private non-synonymous, mt-rRNA, and mt-tRNA variants, and were not included in the table.

b The search was performed on September 13, 2010 following the same strategy described in Bandelt et al. [32], e.g., both ‘A644G mtDNA’ and ‘644A>G mtDNA’ were queried.

c The conservation analysis was performed by comparing *Homo sapiens* mtDNA (GenBank accession no. J01415) to nine different vertebrate species, *Gorilla gorilla* (NC_001645), *Mus musculus* (AY466499), *Bos taurus* (AY526085), *Equus caballus* (EF597513), *Canis lupus chanco* (EU442884), *Canis familiaris* (DQ480502), *Balaenoptera musculus* (NC_001601), *Rana nigromaculata* (AB043889), and *Danio rerio* (NC_002333).

d The column “Haplogroup-specific variant” refers to the presence or absence of the corresponding variants in the world mtDNA phylogeny displayed at http://www.phylotree.org/tree/main.htm (mtDNA tree Build 10; 10 Aug 2009) [29]. In round brackets we indicated the haplogroup status as it defined in that tree.

### mtDNA haplogroup distribution in Chinese LHON patients with m.14484T>C

In order to address a possibility whether there is certain mtDNA haplogroup contributing to the clinical expression of LHON in Chinese patients with m.14484T>C, we analyzed the mtDNA control-region sequences of the 52 probands with m.14484T>C and classified each mtDNA into respective haplogroup (Table S1). All the subjects could be classified into 17 haplogroups based on the control-region sequence motif recognition and matching or near-matching with the published mtDNAs [36] (Table 2). Compared with the pooled Han Chinese population from published sources ([36], [37] and references therein), we found that haplogroups C (*P* = 0.002, OR = 5.614, 95% CI = 2.072–15.208), G (*P* = 0.017, OR = 3.671, 95% CI = 1.345–10.018), M10 (*P* = 0.036, OR = 4.167, 95% CI = 1.209–14.356), and Y (*P* = 0.018, OR = 5.583, 95% CI = 1.521–20.489) had significantly higher frequencies in the LHON subjects. Only one out of the 52 probands with m.14484T>C belonged to haplogroup F. The frequency of haplogroup F (1.9%, 1/52) was significantly lower than that of the Han Chinese from general populations (16.67%, 68/408) (*P* = 0.003, OR = 0.098, 95% CI = 0.013–0.722), which suggested a possibly protective effect of this matrilineal background on LHON (Table 2).

**Table 2:** Haplogroup distribution of 52 Chinese LHON subjects with m.14484T>C.

Haplogroup	No. of subjects with m.14484T>C (%)	No. of Han Chinese from general populations (%) a	*p* value b	Odds Ratio	95% CI
B4	3 (5.77)	55 (13.48)	0.180	0.393	0.118–1.304
B5	5 (9.62)	17 (4.17)	0.090	2.447	0.863–6.937
C	7 (13.46)	11 (2.70)	0.002	5.614	2.073–15.208
D4	6 (11.54)	56 (13.73)	0.830	0.820	0.335–2.009
D5	4 (7.69)	27 (6.62)	0.768	1.176	0.395–3.505
F	1 (1.92)	68 (16.67)	0.003	0.098	0.013–0.722
G	6 (11.54)	14 (3.43)	0.017	3.671	1.345–10.018
M10	4 (7.69)	8 (1.96)	0.036	4.167	1.209–14.355
M12	1 (1.92)	2 (0.49)	0.303	3.980	0.355–44.676
M7b	5 (9.62)	24 (5.88)	0.356	1.702	0.620–4.673
M8a	1 (1.92)	18 (4.41)	0.710	0.425	0.056–3.250
M9a	1 (1.92)	10 (2.45)	1.000	0.780	0.098–6.223
N9a	1 (1.92)	15 (3.68)	1.000	0.514	0.066–3.971
Y	4 (7.69)	6 (1.47)	0.018	5.583	1.522–20.488
Z	1 (1.92)	8 (1.96)	1.000	0.980	0.120–8.000
U	1 (1.92)	3 (0.74)	0.382	2.647	0.270–25.927
R9 excluding F c	1 (1.92)	6 (1.47)	0.571	1.314	0.155-11.132
Other d	0 (0)	60 (14.71)	0.001	-	-

a The Han Chinese from general populations were taken from published sources ([36], [37] and references therein).

b The Fisher exact test (two-tailed) was performed to discern the frequency difference of certain haplogroup between the LHON subjects (N = 52) and Han Chinese from the general populations (N = 408).

c Only mtDNAs belonging to R9b and R9c were considered here, mtDNAs belonging to haplogroup F were not included.

d Those haplogroups that were not observed in the LHON patients but in reported Han Chinese were grouped together.

The complete mtDNA and control-region sequences generated in this study have been deposited in GenBank under accession numbers HM460791-HM460842 and HQ260970-HQ260973.

## Discussion

LHON patients with mutation m.14484T>C exhibited some unique characteristics compared with LHON patients caused by the other primary mutations, such as better visual recovery, obvious gender bias, and higher occurrence in haplogroup J1 in European populations [1]. In this study, we presented an analysis of the largest number of Chinese patients with LHON and m.14484T>C, with an intention to discern potentially pathogenic mutations that were co-segregated with m.14484T>C and to dissect the matrilineal genetic structure of Chinese LHON patients with this mutation. The frequency of mutation m.14484T>C (9.7%) was significantly lower in Chinese LHON patients than that of European patients (20.8%) [9], [15], [23], suggesting racial difference.

Among the eleven probands that were sequenced the entire mtDNA genome, we identified eight private non-synonymous variants, four mt-tRNA variants, and one 16S rRNA variant in the terminal branches of the classification tree (Fig. 2). A web-based search [32] and evolutionary analysis for these private variants showed that none of these variants would enact a synergistic effect with m.14484T>C. It thus seemed that the variable level of penetrance of LHON in these families was modulated by a complex involvement of the effect of primary mutation m.14484T>C, mtDNA background, nuclear genetic background, as well as environmental factor. Although we did not determine the entire mtDNA sequences for all the remaining 41 subjects with m.14484T>C, we would expect that there was no co-segregation of pathogenic mutations with m.14484T>C on the basis of the pattern observed in the 11 families.

The dissection of the matrilineal genetic structure of Chinese LHON subjects with m.14484T>C yielded several interesting findings. First, haplogroup F had a high incidence in South China and the overall frequency of this haplogroup ranged 6%–25.9% across China [36]. In the pooled Han Chinese from general populations, this haplogroup had a frequency of 16.7% (68/408), much higher than what we observed for the LHON subjects with m.14484T>C (1.9%; 1/52). This result was unexpected, but a extremely low occurrence of haplogroup F had been observed in Chinese LHON patients with m.11778G>A (2.2%; 4/182) [15] and in 40 LHON patients harboring m.11778G>A from Thailand (0%; 0/40) [38]. The exact reason for the low occurrence of haplogroup F in Chinese LHON families with m.14484T>C and m.11778G>A remains unclear. An audit for the haplogroup-specific variants for haplogroup F revealed no seemingly useful information for us to deduct the putative role of this genetic background, as the three haplogroup-characteristic polymorphisms (m.249delA in the mtDNA control-region, m.6392T>C in the *MT-CO1* gene, and m.10310G>A in the *MT-ND3* gene) were either located in the non-coding region or caused no amino acid change. Whether there are some interactions between haplogroup F and nuclear genes or environmental factors in these LHON families is an intriguing yet pending puzzle.

Second, haplogroups C, G, Y, and M10 seemed to be more common in Chinese LHON subjects with m.14484T>C than those of Han Chinese from general populations. However, any claim for potential association between these four haplogroups and the risk of LHON should be received with caution and verified by a large sample size. Haplogroup G was found to be possibly associated with the clinical expression of LHON in Chinese families with m.11778G>A in our previous study [15]. However, the penetrance of LHON in the three families with m.14484T>C (,Le616, Le700, and Le1322) belonging to haplogroup G varied substantially (Fig. 1). The putative effect of haplogroup G-specific variant m.4833A>G (p.T122A) on the risk of LHON [15] in these LHON families needs further study. The higher prevalence of haplogroup Y in LHON patients with m.14484T>C was puzzling, as this haplogroup was generally infrequent in East Asian [36]. Among the list of haplgroup Y specific polymorphisms, two non-synonymous variants, m.10398A>G (p.T114A) and m.14178T>C (p.I166V), and one tRNA-Glu variants (m.14693A>G) might deserve some attention. Variant m.10398A>G was reported to be associated with Parkinson disease [39] and bipolar disorder [40] and was said to play a role in mitochondrial matrix pH and intracellular calcium dynamics [41]. However, any speculation for a putative role of this variant or haplogroup Y in Chinese LHON subjects with m.14484T>C should be further tested with more samples and functional assay. In Han Chinese, besides these mtDNAs belonging to haplogroup Y, all mtDNAs belonging to (1) macro-haplogroup M and its subhaplogroups and (2) haplogroups R11, B4c1c, B5 contain m.10398A>G. Therefore, in our LHON patients, the frequency of m.10398A>G could be calculated by adding up the number of lineages belonging to the above haplogroups, then divided by the total number of samples (45/52 = 86.54%). The frequency of m.10398A>G in Han Chinese from general populations could be estimated as 223/408 = 54.66%. The frequency difference of m.10398A>G between the case group and the control group was statistically significant (*P = *0.000005, Fisher's exact test, two-tailed), suggesting that m.10398A>G might act as a potentially confounding factor for m.14484T>C. Again, this result should be treated with caution because of the small sample size of the patients under study.

The higher frequency of haplogroups C and M10 in LHON patients with m.14484T>C was also interesting, as population substructure might not well explain this pattern; but again this observation might be affected by the small sample size of the case group. Note that the four LHON subjects belonging to M10 could be further classified into haplogroup M10a1, which is characterized by one non-synonymous variant m.13135G>A (p.A267T) in the *MT-ND5* gene. A previous study reported that m.13135G>A was associated with familial hypertrophic cardiomyopathy in Chinese [42].

In summary, we presented here the genetic analysis of the largest number of Chinese LHON patients with m.14484T>C to date. We found no pathogenic mutation that was co-segregated with m.14484T>C in all eleven LHON probands that were analyzed for complete mtDNA genome sequences. Noteworthily, the matrilineal genetic structure of the LHON subjects differed from that of Han Chinese from the general populations substantially, with fewer lineages belonging to haplogroup F and higher number of lineages to haplogroups G, C, Y, and M10 in the LHON subjects with m.14484T>C. How and why these mtDNA haplogroups presented such a unique pattern need further study with more samples. Our current study provided an overall matrilineal genetic profile for Chinese LHON patients with m.14484T>C and might be useful for genetic counseling. Extensive analysis of more families would help to further define the risk factors in Chinese patients with m.14484T>C.

## Supporting Information

Table S1 mtDNA sequence variation and haplogroup classification of Chinese families/singleton cases with m.14484T>C and LHON.
(0.15 MB DOC)
