# Supplementary material for: Mitochondrial DNA Sequence Variation and Haplogroup Distribution in Chinese Patients with LHON and m.14484T>C
Source: PLoS One. 2010 Oct 18;5(10):e13426. doi: 10.1371/journal.pone.0013426 (PMC2956641; doi:10.1371/journal.pone.0013426)
Supplement: Table S1 — mtDNA sequence variation and haplogroup classification of Chinese families/singleton cases with m.14484T>C and LHON. (0.15 MB DOC) [file pone.0013426.s001.doc]

Table S1 mtDNA sequence variation and haplogroup classification of Chinese families / singleton cases with m.14484T>C and LHON

| **Sample** | **Haplogroup** | **Sequenced range** ^a^ | **Segment I**  (16000+) | **Segment II**  (all with 73) | **Coding region variations** ^b^ |
| --- | --- | --- | --- | --- | --- |
| Le182 | B4a | 16039-16569 and 1-658 | 169 182C 183C 189 217 239 261 519 | 263 309+CC 315+C 523-524d |  |
| Le603 | B4a | 16015-16569 and 1-907 | 182C 183C 189 217 221 240 261 | 263 309+CC 315+C 750 |  |
| Le1514 | B4a | 16009-16569 and 1-909 | 182C 183C 189 217 221 240 261 | 263 309+CC 315+C 750 |  |
| Le1018 | B5a | 16013-16569 and 1-906 | 140 183C 189 218 266A 519 | 210 263 309+C 315+C 523-524d 709 750 |  |
| Le1233 | B5a | 16015-16569 and 1-863 | 140 183C 189 218 261 266A 519 | 210 263 315+C 709 750 |  |
| Le921 | B5a | 16012-16569 and 1-911 | 140 183C 189 266A 519 | 210 263 309+CC 315+C 523-524d 709 750 |  |
| Le1528 | B5a | 16024-16569 and 1-919 | 182C 183C 189 261 266A 519 | 152 210 263 309d 315+C 523-524d 709 750 |  |
| Le1229 | B5b2c | 16012-16569 and 1-860 | 111 140 162 169+C 182C 183C 189 234 243 463 519 | 94 103 131 263 309+C 315+C 481 523-524d 709 750 |  |
| Le1149 | C | 16021-16569 and 1-832 | 223 298 327 | 234 249d 263 309+C 315+C 489 750 |  |
| Le1507 | C | 16020-16569 and 1-920 | 117 129 223 298 327 519 | 195 228 249d 263 309+C 315+C 489 750 | 14318 |
| Le559 | C | 16035-16569 and 1-661 | 93 129 213 223 298 327 519 | 195 249d 309+C 315+C 489 |  |
| Le865 | C | 19038-16569 and 1-670 | 93 129 213 223 298 327 519 | 195 249d 309+C 315+C 489 |  |
| Le868 | C | 16031-16569 and 1-662 | 129 223 298 327 | 249d 263 315+C 489 523-524d |  |
| ***Le1187*** | C4a | — | 129 192 223 298 327 519 | 249d 263 315+C 489 750 |  |
| Le1337 | C | 16031-16569 and 1-760 | 129 150 209 223 298 327 519 | 195 249d 263 315+C 489 750 |  |
| Le715 | D4 | 16038-16569 and 1-668 | 201 223 362 | 152 263 309+C 315+C 489 | 5178A |
| Le759 | D4 | 16014-16569 and 1-911 | 223 286 362 | 263 309+CC 315+C 489 750 | -5176*Alu*I |
| Le983 | D4 | 16014-16569 and 1-817 | 223 286 362 | 263 309+CC 315+C 489 750 | -5176*Alu*I |
| Le879 | D4 | 16048-16569 and 1-670 | 223 260 292 311 362 | 146 152 263 315+C 489 | -5176*Alu*I, 14668 |
| Le678 | D4a | 16016-16569 and 1-871 | 129 223 256 362 519 | 151 152 263 315+C 489 750 |  |
| Le1184 | D4b1 | 16024-16569 and 1-917 | 185 189d 223 232A 319 362 | 263 315+C 374 489 523-524d 750 |  |
| Le1530 | D5b | 16013-16569 and 1-918 | 172 189 223 362 519 | 150 152 263 309+C 315+C 456 489 681 750 | 14560 |
| ***Le691*** | D5a2a | — | 92 164 172 182C 183C 189 223 266 311 362 | 150 263 315+C 489 750 752 |  |
| ***Le338*** | D5a2a | — | 131A 164 172 182C 183C 189 223 266 362 | 150 152 263 309+C 315+C 489 523-524d 709 750 752 |  |
| ***Le671*** | D5c | — | 189 190 193+2C 223 311 316 356 362 | 150 151 152 263 309+CC 315+C 489 750 |  |
| Le785 | R9c | 16017-16569 and 1-866 | 157 256 304 335 | 249d 263 309+CC 315+C 750 |  |
| Le1118 | F1a1 | 16029-16569 and 1-913 | 129 162 172 304 399 519 | 249d 263 309+C 315+C 523-524d 750 |  |
| Le1 | G | 16025-16569 and 1-876 | 223 362 519 | 263 309+C 315+C 489 593 709 750 | +5176*Alu*I, 14420, 14569 |
| ***Le1322*** | G2b2 | — | 223 260 291 362 519 | 143 204 263 315+C 489 709 750 | +5176*Alu*I, 14569 |
| ***Le1123*** | G1a1 | — | 223 325 362 463 519 | 150 263 489 709 750 |  |
| Le752 | G2a | 16023-16569 and 1-875 | 86 129 223 278 362 368 526 | 263 309+C 315+C 489 709 750 |  |
| ***Le616*** | G2a1 | — | 86 129 223 278 362 368 526 | 263 309+C 315+C 489 709 750 |  |
| ***Le700*** | G2a1 | — | 176 223 227 278 293C 294(*h*) 362 | 263 309+CC 315+C 318 489 709 750 |  |
| ***Le1304*** | M10a1a | — | 93 129 223 311 357 497 | 263 309+C 315+C 489 523-524d 573+3C 709 |  |
| Le191 | M10a1 | 16008-16569 and 1-870 | 93 129 223 311 357 497 | 143 263 315+C 489 523-524d 573+6C 709 750 |  |
| Le567 | M10a1 | 16051-16569 and 1-619 | 193 223 311 357 497 | 146 152 263 309+C 315+C 489 523-524d 573+4C | 14502 |
| Le405 | M10a1 | 16038-16569 and 1-614 | 93 129 223 266 311 357 497 | 263 309+C 315+C 489 523-524d 573+4C |  |
| Le1550 | M12 | 16014-16569 and 1-903 | 189 223 234 290 362 | 125 127 128 263 315+C 318 489 513-514d 750 | 14569 |
| Le1126 | M7b1 | 16013-16569 and 1-817 | 129 189 192 223 297 | 150 199 238 263 309+C 315+C 489 750 |  |
| Le1251 | M7b1 | 16013-16569 and 1-751 | 129 192 223 297 | 150 199 263 309+CC 315+C 489 524+AC 750 |  |
| Le1364 | M7b1 | 16012-16569 and 1-792 | 129 189 192 223 297 | 150 199 238 263 309+C 315+C 489 750 |  |
| Le987 | M7b1 | 16015-16569 and 1-791 | 129 192 223 297 | 150 195 199 263 309+C 315+C 489 750 |  |
| Le1607 | M7b1 | 16013-16569 and 1-909 | 129 189 192 223 297 | 150 199 238 263 309+C 315+C 489 750 |  |
| Le1578 | M9a | 16017-16569 and 1-852 | 223 234 316 362 | 146 153 263 309+C 315+C 489 750 | 14308 |
| ***Le789*** | M8a | — | 86 184 223 298 319 | 146 263 315+C 489 750 |  |
| Le1379 | N9a | 16022-16569 and 1-909 | 223 257A 261 | 150 263 309+C 315+C 750 |  |
| Le607 | U2e | 16012-16569 and 1-809 | 51 126 129C 189 256 298 362 519 | 152 217 263 315+CC 340 508 524+AC 573+CC 750 |  |
| Le1350 | Y1 | 16012-16569 and 1-911 | 126 231 266 519 | 146 263 309+C 315+C 750 |  |
| Le1103 | Y1 | 16032-16569 and 1-911 | 126 224 231 266 519 | 146 263 309+C 315+C 750 |  |
| Le697 | Y2 | 16011-16569 and 1-911 | 126 231 311 519 | 195 263 309+CC 315+C 482 523-524d 750 |  |
| ***Le708*** | Y2 | — | 126 231 311 | 263 315+C 482 523-524d 644 750 |  |
| Le1327 | Z | 16013-16569 and 1-856 | 185 223 260 298 | 152 214 249d 263 315+C 417 489 709 750 |  |

Note – Sequence variation was scored relative to the revised Cambridge reference sequence (rCRS) [1]. Suffixes A, G, C, and T indicated transversions, “d” and “+” indicated deletions and insertions, respectively. Indels (insertion and deletion) were recorded at the last possible site. “+” and “－” denote dthe absence and presence of the restriction site, respectively. The heteroplasmic variant was marked by “(*h*)”. These samples that were analyzed for the entire mtDNA sequences were marked in italic and bold font

^a^ All the samples were sequenced for regions 16051-16569 and 1-614; some samples were amplified using primer pair (L15594: 5’-CGCCTACACAATTCTCCGATC-3’ and H901: 5’-ACTTGGGTTAATCGTGTGACC-3’) that was reported in [2] and the determined sequences covered a longer region. We listed all the variants in these sequences. This is the reason why some samples contained variation at positions 709, 750, 752 that was beyond region 1-614

^b^ The coding region variations were screened by using sequencing and/or RFLP analyses

**Supplemental references**

1. Andrews RM, Kubacka I, Chinnery PF, Lightowlers RN, Turnbull DM, et al. (1999) Reanalysis and revision of the Cambridge reference sequence for human mitochondrial DNA. Nat Genet 23:147

2. Yao Y-G, Ogasawara Y, Kajigaya S, Molldrem JJ, Falcão RP, et al. (2007) Mitochondrial DNA sequence variation in single cells from leukemia patients. Blood 109:756-762
